# Genomic Common Data Model for Seamless Interoperation of Biomedical Data in Clinical Practice: Retrospective Study

**DOI:** 10.2196/13249

**Published:** 2019-03-26

**Authors:** Seo Jeong Shin, Seng Chan You, Yu Rang Park, Jin Roh, Jang-Hee Kim, Seokjin Haam, Christian G Reich, Clair Blacketer, Dae-Soon Son, Seungbin Oh, Rae Woong Park

**Affiliations:** 1 Department of Biomedical Sciences Ajou University Graduate School of Medicine Suwon Republic of Korea; 2 Department of Biomedical Informatics Ajou University School of Medicine Suwon Republic of Korea; 3 Department of Biomedical Systems Informatics Yonsei University College of Medicine Seoul Republic of Korea; 4 Department of Pathology Ajou University Hospital Suwon Republic of Korea; 5 Department of Thoracic & Cardiovascular Surgery Ajou University Hospital Suwon Republic of Korea; 6 IQVIA Durham, NC United States; 7 Department of Epidemiology Janssen Research and Development Titusville, NJ United States; 8 Samsung Genome Institute Samsung Medical Center Seoul Republic of Korea; 9 Department of Pharmacy Kangwon University Chuncheon Republic of Korea

**Keywords:** high-throughput nucleotide sequencing, databases, genetic, multicenter study, patient privacy, data visualization

## Abstract

**Background:**

Clinical sequencing data should be shared in order to achieve the sufficient scale and diversity required to provide strong evidence for improving patient care. A distributed research network allows researchers to share this evidence rather than the patient-level data across centers, thereby avoiding privacy issues. The Observational Medical Outcomes Partnership (OMOP) common data model (CDM) used in distributed research networks has low coverage of sequencing data and does not reflect the latest trends of precision medicine.

**Objective:**

The aim of this study was to develop and evaluate the feasibility of a genomic CDM (G-CDM), as an extension of the OMOP-CDM, for application of genomic data in clinical practice.

**Methods:**

Existing genomic data models and sequencing reports were reviewed to extend the OMOP-CDM to cover genomic data. The Human Genome Organisation Gene Nomenclature Committee and Human Genome Variation Society nomenclature were adopted to standardize the terminology in the model. Sequencing data of 114 and 1060 patients with lung cancer were obtained from the Ajou University School of Medicine database of Ajou University Hospital and The Cancer Genome Atlas, respectively, which were transformed to a format appropriate for the G-CDM. The data were compared with respect to gene name, variant type, and actionable mutations.

**Results:**

The G-CDM was extended into four tables linked to tables of the OMOP-CDM. Upon comparison with The Cancer Genome Atlas data, a clinically actionable mutation, p.Leu858Arg, in the *EGFR* gene was 6.64 times more frequent in the Ajou University School of Medicine database, while the p.Gly12Xaa mutation in the *KRAS* gene was 2.02 times more frequent in The Cancer Genome Atlas dataset. The data-exploring tool GeneProfiler was further developed to conduct descriptive analyses automatically using the G-CDM, which provides the proportions of genes, variant types, and actionable mutations. GeneProfiler also allows for querying the specific gene name and Human Genome Variation Society nomenclature to calculate the proportion of patients with a given mutation.

**Conclusions:**

We developed the G-CDM for effective integration of genomic data with standardized clinical data, allowing for data sharing across institutes. The feasibility of the G-CDM was validated by assessing the differences in data characteristics between two different genomic databases through the proposed data-exploring tool GeneProfiler. The G-CDM may facilitate analyses of interoperating clinical and genomic datasets across multiple institutions, minimizing privacy issues and enabling researchers to better understand the characteristics of patients and promote personalized medicine in clinical practice.

## Introduction

### Background

Recognition of the importance of clinical next-generation sequencing (NGS) in precision medicine has had a profound impact on improving medical care [1-3]. Patients’ sequencing data are currently generated through relatively large-scale projects aimed at exploring the role of clinical NGS in precision medicine conducted by organizations such as the American Association for Cancer Research Project GENIE [4] and the China Precision Medicine Initiative [5]. However, genomic data are considered to be privacy sensitive and potentially reidentifiable, which raises concerns about transmitting and sharing patient-level data outside of host institutions for collaborative research [6]. In addition, genomic sequencing data of subjects in a predefined cohort cannot reflect the full diversity of the entire population at the point of care, which limits the practical application of the data for research purposes [7].

There has been a recent widespread effort to collect genomic information on patients in clinical practice through routine laboratory tests by the UK Biobank [8] and Geisinger Health System [9]. Since March 2017, the South Korea government has provided conditional insurance for an NGS technology-based cancer gene panel [10], which is expected to lead to rapid accumulation of clinical sequencing data in each hospital. However, the vocabulary and structure of these datasets are not standardized, which makes it difficult to conduct appropriate multicenter or comparative analyses for clinical decision making [11]. This lack of standardization can be overcome by using the common data model (CDM), which applies the same data structure to run an identical analysis code for each data holder [12]. For example, the Informatics for Integrating Biology and the Bedside is a clinical data warehouse platform comprising genetic data that adopts the CDM to support the distributed research network [13,14], an infrastructure for novel internet-based strategies that allows researchers to use retrospective multicenter data in a CDM (in contrast to single-center or cloud-based research) without exporting the protected personal health information. Researchers can combine the results of an analysis code run over the network to generate a refined clinical hypothesis [12,15]. To date, the distributed research network has been adopted by global research collaboration groups, including the Observational Health Data Sciences and Informatics (OHDSI) consortium [16]. The Observational Medical Outcomes Partnership (OMOP) CDM, now in version 6.0, was developed by the OHDSI consortium and includes clinical data from over 20 countries, with information of 1.5 billion patients transformed to date.

### Prior Work

Due to the nature and extraordinary complexity of sequencing data, it is challenging to effectively describe and interpret the status of sequence alterations [17]. Furthermore, sequencing data were applied in the clinical domain of NGS relatively later than other types of genomic tests; hence, the analytical process has not been standardized [18]. To improve the efficiency of data processing, sequencing data should be managed using standardized structures and semantics. Although several standard models for genomic data have been introduced to date, they have limited applicability. For example, the standard for non-NGS–specific data models, including the minimum information about a microarray experiment [19] for DNA microarray analysis, the tissue microarray object model [20] for tissue microarray analysis, and the proteomics experiment data repository [21] for proteomics, cannot be properly adopted for sequencing data. Although the minimum information about a high-throughput nucleotide sequencing experiment was developed as a data model specific for sequencing data, it requires experimental processing data and detailed analytical protocols to enable researchers to reproduce the analysis [22].

### Aim

Given the limitations outlined above, the objective of this study was to create a genomic data CDM (G-CDM) for use in the distributed research network. To address patient privacy issues and support the diversity of genomic data such as ethnicity, the OMOP-CDM used in the OHDSI consortium was chosen for this study for expansion. Furthermore, we validated the feasibility of the model by exploring the difference in genomic data retrieved from public databases and clinical practice.

## Methods

### Construction of the Genomic Data Common Data Model

The proposed G-CDM was developed by extending the OMOP-CDM to achieve the seamless management of clinical sequencing data through a structured database model. Clinical information such as basic patient background (eg, sex and age), clinical diagnosis, procedures, or specimen type was stored in existing tables of the OMOP-CDM. We further reviewed other genomic data models and clinical sequencing reports to design additional tables for describing and interpreting sequence alterations occurring in target genes. There are various types (>50) of public cancer databases describing variants, including comprehensive cancer projects, resources, and cancer type-specific databases [23]. According to our inclusion and exclusion criteria (Multimedia Appendix 1), we selected datasets from The Cancer Genome Atlas (TCGA), Catalogue of Somatic Mutations in Cancer, and International Cancer Genome Consortium for review and reference, to define the method of sequence alteration description. The data quality of these representative databases has been validated through many studies and papers. The database TCGA provides large-scale datasets of genomic alterations, including insertions/deletions (INDELs) or single nucleotide polymorphisms (SNPs), discovered in over 30 human tumor types to generate comprehensive profiles of cancer genomics [24]. The database Catalogue of Somatic Mutations in Cancer provides somatic mutations across 1,391,372 tumor samples encompassing 5,977,977 coding mutations as of August 2018 [25], while the database International Cancer Genome Consortium provides the datasets of oncogenic mutations of 50 different cancer types to support large-scale studies [26,27]. We excluded the databases built based on non-NGS techniques or cancer type–specific databases from referencing. The ISO20428 document, which is a standard format for reporting sequencing results, was reviewed to design columns for variant annotation (Multimedia Appendix 2). To guarantee interoperability of the data, standard terminologies were adopted in the G-CDM [28,29]. The name of a human gene, a key factor in sequencing data, was fixed according to the nomenclature of the Human Genome Organisation Gene Nomenclature Committee, which currently contains and maintains approximately 41,000 unique gene symbols. In addition, the Human Genome Variation Society nomenclature was adopted to standardize the manner of describing sequence alterations in each gene at both the DNA and protein level. Although either one- or three-letter abbreviations are permitted in the Human Genome Variation Society nomenclature, we propose expressing the amino acid by its three-letter code only to permit seamless data analysis for widespread research (Multimedia Appendix 2).

### Data Structure of the Genomic Data Common Data Model

**Figure 1:**
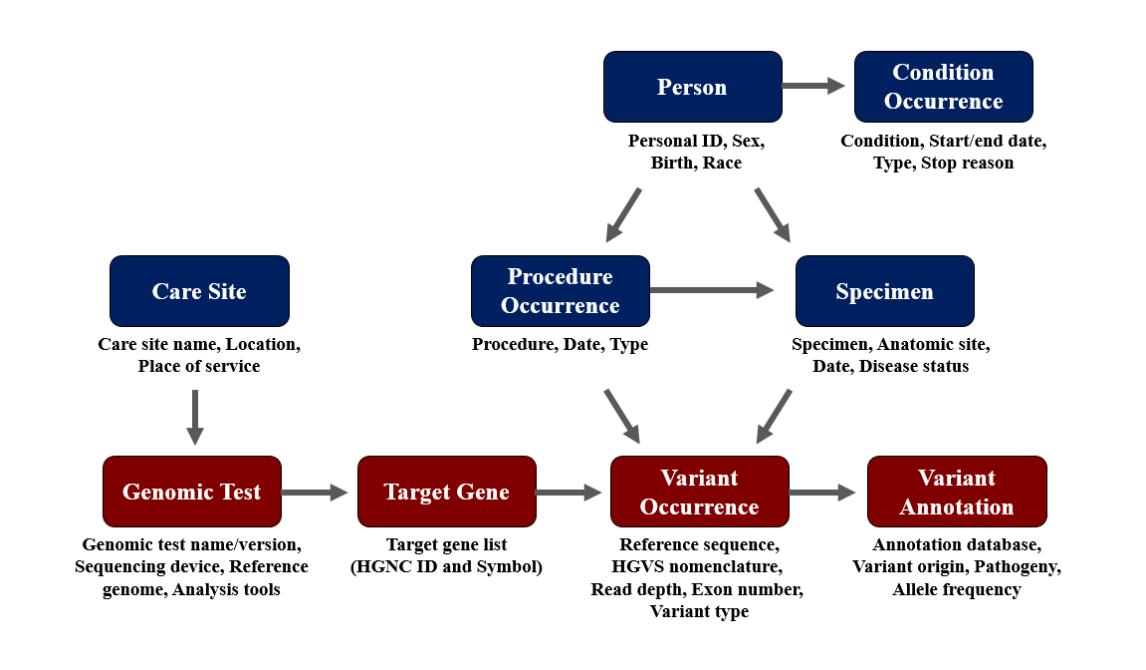
Schematic diagram of the relationship between tables composing the genomic common data model. Tables in red (“Genomic_Test,” “Target_Gene,” “Variant_Occurrence,” and “Variant_Annotation”) are those storing genomic sequencing data and processes, whereas tables in blue (“Person,” “Condition_Occurrence,” “Procedure_Occurrence,” “Specimen,” and “Care_Site”) are those already existing in the Observational Medical Outcomes Partnership-common data model and store clinical data directly linked to the “Variant_Occurrence” and “Genomic_Test” tables. ID: identification; HGVS: Human Genome Variation Society; HGNC: Human Genome Organisation Gene Nomenclature Committee.

To link clinical data in the OMOP-CDM, the following information on each patient with NGS data was stored in a separate corresponding table: Person, Condition_Occurrence (diagnosis), Procedure_Occurrence, Specimen, and Care_Site (Figure 1). The Person table included personal patient information such as individual identification, sex, age, and race. The Condition_Occurrence table contained information on the patient’s condition or diagnosis, including the disease such as “lung cancer” or condition type such as “primary condition.” The Procedure_Occurrence table included information on how the specimen used for NGS was obtained and the name of the genomic test conducted for a patient. The Specimen table included information on the specimen used for the genomic test, such as “target” (tumor tissue) and “reference” (normal tissue), along with specimen type, including paraffin-embedded slide, the date the specimen was obtained, and the anatomical site of the specimen. The Care_Site table included information on the site at which the genomic test was conducted.

In addition to these five tables, we expanded the model to be linked to four other tables containing information related to the sequencing data: (1) the Genomic_Test table included the test name, version, sequencing device, analytical tools, and reference databases, with a care site identification column; (2) the Target_Gene table contained a list of genes targeted by the genomic test following Human Genome Organisation Gene Nomenclature Committee nomenclature for standardized gene symbols; (3) the Variant_Occurrence table included descriptive information about the variants of target genes; and (4) the Variant_Annotation table included information on each variant and the clinical interpretation thereof, such as annotation database name, variant origin such as somatic or germline, pathogenicity of the variant, allele frequency, and medication.

Procedure identification for conducting sequencing, specimen identification of both the target and reference specimens, and target gene identification were included as foreign keys to link the information in the Procedure, Specimen, and Target_Gene tables. Data on reference sequence, reference SNP identification, Human Genome Variation Society nomenclature at both the DNA and protein levels, read depth, exon number, and variant type of both structural DNA and functional proteins were stored as variant description parameters. Detailed schemes and descriptions of each column and table used in the genomic extension model are provided in Multimedia Appendices 3 and 4.

### Data Description

The Ajou University School of Medicine (AUSOM) database consists of electronic medical record data of patients who underwent NGS-based cancer panel screening of the tumor tissue between June 2017 and August 2018 at Ajou University Hospital, including 92 patients with lung adenocarcinoma and 22 patients with lung squamous cell carcinoma. Public sequence alteration data of the lung cancer cohort Pan-Lung Cancer study of TCGA [30] were obtained from the Memorial Sloan-Kettering Cancer Center cBioPortal [31].

The overall processes of NGS conducted at Ajou University Hospital and the TCGA database are detailed in Multimedia Appendix 5. Two representative differences between the sequencing pipelines of the two databases are the number of genes and the composition of variant types targeted in the test. For example, in the cancer panel of AUSOM, 49 cancer-related genes were targeted for sequencing, while the TCGA data were harvested using whole-exome sequencing with 16,896 genes. Thus, for development and testing of the proposed G-CDM, we selected 1060 patients from TCGA with available variant data of the 49 target genes selected in the AUSOM panel (Table 1).

**Table 1:** Description of data used to build the genomic common data model and to validate the data model.

Variable		AUSOM^a^ (N=114), n (%)	TCGA^b^ (N=1060), n (%)
**Age (years)**			
	≤49	7 (6.1)	44 (4.2)
	50-59	26 (22.8)	163 (15.4)
	60-69	41 (36.0)	310 (29.2)
	70-79	35 (30.7)	317 (29.9)
	≥80	5 (4.4)	56 (5.2)
	Unknown	0 (0.0)	170 (16.0)
**Gender**			
	Male	64 (56.1)	628 (59.0)
	Female	50 (43.9)	429 (41.0)
	Unknown	0 (0.0)	3 (0.2)
**Pathology**			
	Lung adenocarcinoma	92 (80.7)	603 (56.9)
	Lung squamous carcinoma	22 (19.3)	457 (43.1)
**Cancer stage**			
	Stage I	78 (68.4)	526 (49.6)
	Stage II	16 (14.0)	286 (27.0)
	Stage III	18 (15.8)	184 (17.4)
	Stage IV	0 (0.0)	36 (3.4)
	Unknown	2 (1.8)	28 (2.6)

^a^AUSOM: Ajou University School of Medicine.

^b^TCGA: The Cancer Genome Atlas.

The variant types, including SNPs, INDELs, multinucleotide polymorphisms (MNPs), copy number variants (CNVs), and translocations, were explored in the AUSOM database, whereas only SNPs and INDELs were identified in the TCGA database. Information on clinical characteristics such as age, sex, and disease status and genomic alterations such as variant type, DNA and protein level changes, and functional impact were used to compare the AUSOM and TCGA databases.

### Study Design

Sequencing data of the TCGA database, which was licensed by Yonsei University for use, and of the AUSOM database were transformed into the G-CDM at Yonsei University and Ajou University, respectively. To execute the transformation process, the Structured Query Language (SQL) script in Microsoft SQL Server 2017 was used as the relational database backend for storage and querying the sequencing data. The G-CDM database was built using the Intel Xeon CPU E5-2596 v4 2.20 GHz, Java v.1.8.0, R v.3.5.1, and DBMS SQL Server 2017 at Ajou University, while the Intel Xeon Gold 6132 CPU 2.60 GHz, Java v.1.8.0, R v.3.4.4, and DBMS SQL Server 2017 were used at Yonsei University.

After extracting parameters of interest for a cohort of patients by using a Condition_Occurrence table, the genetic information of the patients was summarized in each of the two institutions. Owing to the restrictions on exporting the original clinical sequencing data in the AUSOM database outside the hospital, the two institutions gathered and compared only the descriptive statistical analysis results to compare the two sequencing databases in further research.

The data visualization tool “GeneProfiler” was developed to run based on the G-CDM as a demonstration that the standardized structure and vocabulary system can serve as a usable medium for performing distributed research by allowing genomic analysis with an identical code. To validate the feasibility of the G-CDM as a storage system and analysis medium, the differences in sequencing data between the AUSOM and TCGA databases were explored. The background profile of variants was described based on several aspects such as gene names, variant types, and disease subtypes. Representative actionable mutations for patients with non-small cell lung cancer (NSCLC) tend to occur in the *EGFR*, *KRAS*, *PIK3CA*, *BRAF*, and *NRAS* genes according to National Comprehensive Cancer Network guidelines [32,33]. Therefore, the proportions of actionable mutations in these five genes were compared between the two databases and between the subtypes of lung cancer.

### Data Visualization Tool

**Figure 2:**
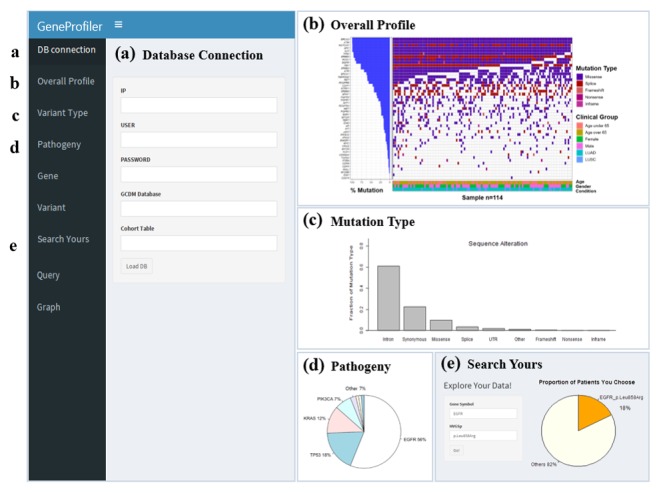
Data visualization tool for clinical sequencing data holders who converted their genomic data into genomic CDM. Users can (a) connect their genomic CDM database; (b) get analysis plots such as an overall profile, (c) mutation type, and (d) pathogeny of variants; and (e) search the proportion of patients with gene name and variant information. CDM: common data model.

We developed a new data visualizing tool called “GeneProfiler” using the R Shiny package to facilitate the utility and accessibility of the G-CDM. After converting genomic data into the G-CDM, the data can be visualized by simply connecting the database with the graphic user interface (Figure 2). As users link their database into “GeneProfiler,” this tool automatically provides the descriptive statistics as several plots and tables. “GeneProfiler” includes action buttons to generate plots of overall variant profiles, proportion of certain mutation types, and proportion of genes with actionable mutations. Users can also freely explore the proportion of patients with mutations in specific genes or specific variants and can download the results as a plot or table to conduct distributed research. After downloading result tables of several databases from GeneProfiler, users can generate graphs comparing these databases by uploading the merged tables (Multimedia Appendix 6). The R Shiny code of “GeneProfiler” was uploaded and is open to the public in GitHub [34].

### Statistical Analysis

Descriptive analysis was performed using frequencies for categorical variables. Genomic characteristics were compared between the two databases using a chi-squared test, and values of *P*<.05 were considered statistically significant. The R program version 3.5.1 was used for data preprocessing and statistical analysis. A mutation waterfall plot was created using “GenVisR,” an R package available via Bioconductor [35], which also provided the proportions of genes, variant types, and specific variants using the R Shiny tool developed in this study.

### Ethics Statement

This study was approved by the institutional review board at Ajou University Hospital of Korea (IRB approval number: AJIRB-MED-MDB-18-390).

## Results

### Data Comparison for Model Validation

To confirm the differences between the AUSOM and TCGA databases, the summary results of the sequencing data such as the gene, variant type, and disease subtypes were gathered and compared. We characterized the biological background of total variants in both databases for variant types, with DNA-level structural variants classified as “sequence alteration” and protein functional types classified as “variant feature.” Among the SNPs, insertions, and deletions, the most frequent structural variant type was SNPs, accounting for >80% of total variants in both databases (Multimedia Appendix 7). However, the functional types of the variants, including missense, nonsense, frameshift, inframe, and splice variants, showed different frequencies between the databases (all *P*<.001), with intron and synonymous variants being most frequent in the AUSOM database (combined frequency of 83%) and missense variants being the most frequent in the TCGA database (73%; Multimedia Appendix 7).

**Figure 3:**
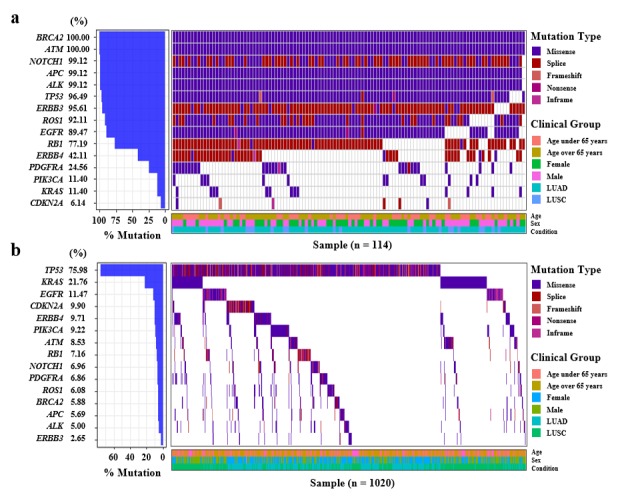
Waterfall plot describing the variant profile of the top 10 genes in (a) Ajou University School of Medicine and (b) The Cancer Genome Atlas databases. Each row represents gene symbols ordered by their frequency of variants with different colors indicating different variant types. Columns represent each patient with only one sample per patient. The bar graph on the left corresponds to the frequency of variants in each gene. Clinical groups such as age, sex, and condition are shown in the bottom box. LUAD: lung adenocarcinoma; LUSC: lung squamous cell carcinoma.

A waterfall plot was created in both the AUSOM and TCGA databases, which focused only on protein-altering variants such as missense, nonsense, frameshift, inframe, and splicing variants to obtain a variant profile (Figure 3; Multimedia Appendix 8). The 15 genes as a union of the top 10 genes in each database were selected as targets for overall profiling. In the AUSOM database, the top 10 genes had a variant frequency > 75% among patients with lung cancer, whereas only one gene, *TP53*, had a variant frequency > 25% in the TCGA database. In particular, *EGFR* variants showed very different frequencies in the AUSOM and TCGA databases (89.5% and 11.5%, respectively). All 15 genes had different proportions of variants in the two databases (all *P*<.001). Although the ranking of genes with high frequencies of variants differed between databases; the most frequent variant type was a missense variant in both databases (Figure 3).

In contrast, comparison of the waterfall plot of all 49 genes targeted in the cancer panel of the AUSOM database to that of the same gene set of the TCGA database showed a higher frequency of frameshift and nonsense type variants than splice type variants in the TCGA data, although the ranking of genes with more variants still differed between the two databases (Multimedia Appendix 8). Exploration of the CNVs in AUSOM showed that *RET* was the gene with the most frequent CNVs, specifically due to amplification (Multimedia Appendix 8).

### Comparison of Actionable Mutations for Model Validation

**Figure 4:**
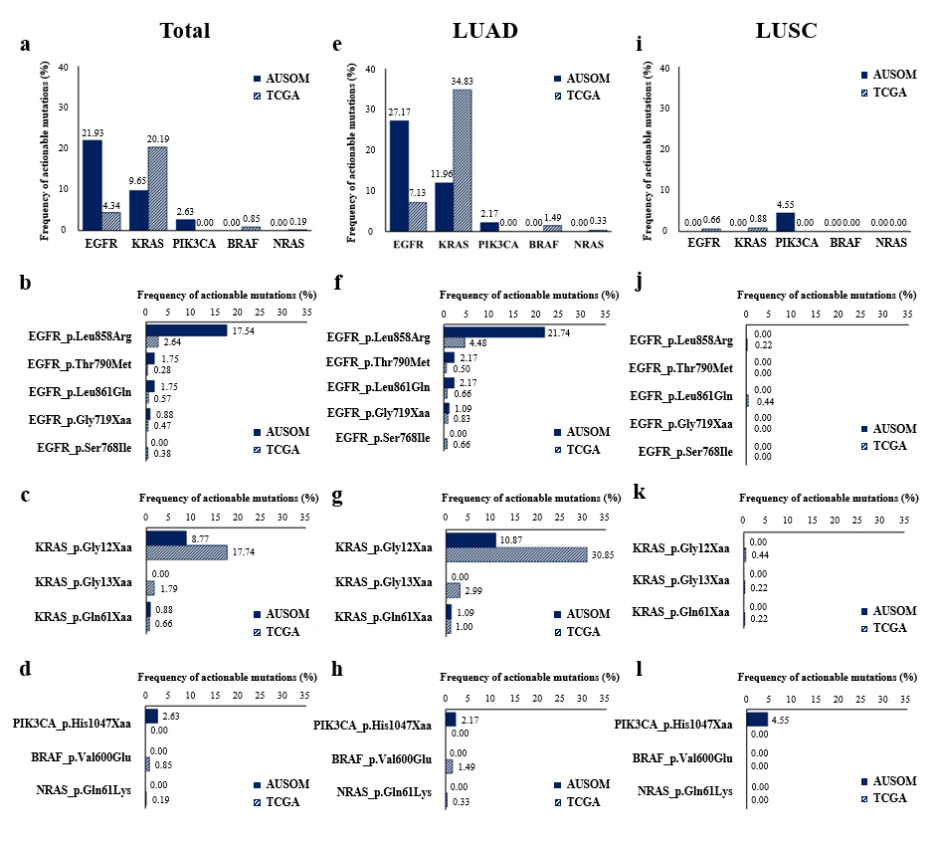
Frequencies of actionable mutations detected in the sequencing process between the AUSOM and TCGA databases. Frequency is shown according to the (a, e, i) level of five selected genes and (b, f, j) actionable mutations in EGFR, (c, g, k) KRAS, and (d, h, l) others such as PIK3CA, BRAF, and NRAS. Frequency is also shown according to patient groups: (a-d) total, (e-h) lung adenocarcinoma, and (k-l) lung squamous cell carcinoma. AUSOM: Ajou University School of Medicine; TCGA: The Cancer Genome Atlas; LUAD: lung adenocarcinoma; LUSC: lung squamous cell carcinoma.

An actionable mutation is a specific genomic event that potentially affects a patient’s response to a targeted therapy [36]. Of the five representative actionable mutations for NSCLC examined (*EGFR*, *KRAS*, *PIK3CA*, *BRAF*, and *NRAS*), *EGFR* showed the greatest frequency of variants in the AUSOM database (21.9%), while *KRAS* showed the greatest frequency of variants in the TCGA database (20.2%; Figure 4a). In particular, the point mutation p.Leu858Arg in *EGFR* was found in 17.5% of the patients, followed by p.Thr790Met (1.8%) in the AUSOM database (Figure 4b). Point mutations in the *KRAS* gene, such as p.Gly12Xaa and p.Gly13Xaa, were more frequent in the TCGA database (20.2%) than in the AUSOM database (9.7%; Figure 4a,c). In addition, patients with lung adenocarcinoma (Figure 4e-h) tended to have more actionable mutations than those with lung squamous cell carcinoma (Figure 4i-l).

## Discussion

### Overview

We developed a new data model for clinical sequencing data, which was applied using sequencing data of patients with lung cancer from two different databases, AUSOM and TCGA, which were transformed into an identical format for the G-CDM. To evaluate the feasibility of the G-CDM, the composition of the datasets was compared with regard to the frequency of a gene name and variant types in which a sequence alteration occurred and to the prevalence of actionable mutations. Moreover, we developed novel user-friendly software—GeneProfiler—for visualization of clinical sequencing data.

### Interpretation of the Principal Results

The first result obtained by comparison of the databases transformed in a standardized form for the G-CDM was the clear difference in the composition of the sequencing data between TCGA, a controlled research-oriented database, and AUSOM, an actual clinical practice database. This difference suggested a difference in variant frequencies and types between the two databases. Indeed, the total number of variants per patient was much higher for the AUSOM database than for the TCGA database, whereas the frequency of variants differed according to the variant type considered. Comparison of actionable mutations in five genes of NSCLC showed a much higher mutation frequency of *EGFR* in the AUSOM database (a cohort of Asian patients) than in the TCGA database (a cohort of American patients). This finding is in line with previous knowledge that Asian patients with NSCLC have a higher prevalence of *EGFR* mutations than Americans [32,37]. In contrast, actionable mutations in the *KRAS* gene were less prevalent in patients in the AUSOM database than in those in the TCGA database, which is also consistent with previous knowledge that Asian populations have a much lower rate of mutations in *KRAS* than non-Asian populations with NSCLC [32,37].

The second key result of this study is conduct of a multicenter research through internet-based sharing of analysis codes with CDM-based conversion of databases from different institutions. This is meaningful because the distributed research was conducted with genomic data that had not been previously verified. Such distributed research would be a useful strategy to address the problem of limited data integration due to privacy issues of clinical sequencing data.

Moreover, because data from the TCGA database were generated relatively earlier than those in the AUSOM database, the sequencing equipment or bioinformatics method may have caused the observed differences. These differences between the databases further emphasize the importance of analyzing data obtained from multiple clinical sites together with research-driven public data to obtain a higher level of representative evidence from diverse populations. Both genomic data models and intermediate results should be shared as widely as possible to promote clinical advances by overcoming the current challenges of unstructured and siloed data environments that lead to a lack of interoperability [38]. Our proposed OMOP-CDM extension model was developed by referencing the OHDSI distributed research network, because existing models such as the HL7 reference information model are not suitable for internet-based research and have limited practical use [39,40].

In the process of modeling the structure of the G-CDM, two specimen identifications were allocated in the Variant_Occurrence table, because recent methods of NGS testing in cancer patients tend to be based on a comparison of normal and tumor tissues simultaneously from the same individual. In cases of patients with a congenital disease, there is an option to fill out this field with only single-specimen identification. The contents of annotation to a variant can also differ according to the type or version of the annotation databases used in the annotation process. For this reason, the Variant_Annotation table was separated from the Variant_Occurrence table to allow for subsequent updating of diverse or new interpretations.

### Limitations

Genomic data are generated using highly complicated sequencing pipelines and analytical processes; consequently, NGS data have inherent limitations in terms of data quality and reliability. Although we compared the sequencing pipelines and analytical processes used to generate the sequencing data of both the AUSOM and TCGA databases, we were unable to confirm the detailed parameters and options used in each process. Thus, the differences between the two databases found in this study should be interpreted considering the possibility that the data may have been generated by dissimilar methods and criteria.

Moreover, the clinical NGS data used in this study were generated in the clinical practice of Ajou University Hospital within the last 2 years. Given the recent time frame, mortality was rare among these patients; thus, we were not able to perform survival analysis by leveraging both genomic data and clinical data.

The G-CDM, as a common data structure and vocabulary system, minimizes privacy issues when conducting multicenter studies by integrating statistical results of the same analysis code rather than sharing the clinical sequencing data directly. However, when the G-CDM is used for repeated queries with a malicious purpose, there is concern for compromising the privacy of the individual, even if the queries target only the aggregated statistics. The G-CDM can be complemented by inhibiting reidentification attacks, as proposed in previous studies related to the mitigation of privacy risks, through limiting response to a query targeting a unique individual or through introduction of noise into the original data [41,42].

### Conclusions

We propose the distributed research network–based G-CDM as a starting point for a broad community discussion on genomic data–based precision medicine. Based on the G-CDM developed in this study, the data validation process identified differences between the clinical NGS data derived from a clinical practice and those derived from prospective research. We believe that the construction and adoption of this standard data model will facilitate the usefulness of clinical NGS data.

## Appendix

Multimedia Appendix 1 Inclusion and exclusion criteria for databases used for review and reference to define the method of sequence alteration description.

Multimedia Appendix 2 Architecture of the genomic common data model (G-CDM). Conceptualized description of the genomic models, databases, and nomenclature referenced during the G-CDM development process.

Multimedia Appendix 3 Genomic common data model (G-CDM) entity-relationship diagram as an extension of the Observational Medical Outcomes Partnership (OMOP)-CDM. Tables with genomic data (red) and clinical data (blue) are linked. Not all columns composing each table are shown for clarity and conciseness.

Multimedia Appendix 4 Description of the genomic common data model (G-CDM) specifications as an extension of the Observational Medical Outcomes Partnership (OMOP)-CDM.

Multimedia Appendix 5 Next-generation sequencing pipelines used at Ajou University School of Medicine (AUSOM) and for the data collected in The Cancer Genome Atlas (TCGA).

Multimedia Appendix 6 GeneProfiler for conducting distributed research. (a) Using the "Query" tab, an identical analysis query can be submitted to datasets of different institutions. (b) Using the "Graph" tab, a comparative graph can be generated by inputting a merged table containing the analysis results of several institutions.

Multimedia Appendix 7 Characterization of the biological background of lung cancer, including lung adenocarcinoma (LUAD) and squamous cell carcinoma (LUSC). The fractions of (a) structural mutations and (b) functional mutations are shown according to mutation type between the Ajou University School of Medicine (AUSOM) and The Cancer Genome Atlas (TCGA) datasets.

Multimedia Appendix 8 Overall mutation profile of lung cancer patients for (a-b) total targeted genes in the Ajou University School of Medicine (AUSOM) and The Cancer Genome Atlas (TCGA) databases and (c) copy number variations in AUSOM.

## Abbreviations

AUSOM: Ajou University School of Medicine
CDM: common data model
CNV: copy number variant
G-CDM: genomic common data model
INDEL: insertion/deletion
NGS: next-generation sequencing
OHDSI: Observational Health Data Sciences and Informatics
OMOP: Observational Medical Outcomes Partnership
SNP: single nucleotide polymorphism
SQL: structured query language
TCGA: The Cancer Genome Atlas
