# Supplementary figures and images for: Genomic Common Data Model for Seamless Interoperation of Biomedical Data in Clinical Practice: Retrospective Study
Source: J Med Internet Res. 2019 Mar 26;21(3):e13249. doi: 10.2196/13249 (PMC6454347; doi:10.2196/13249)

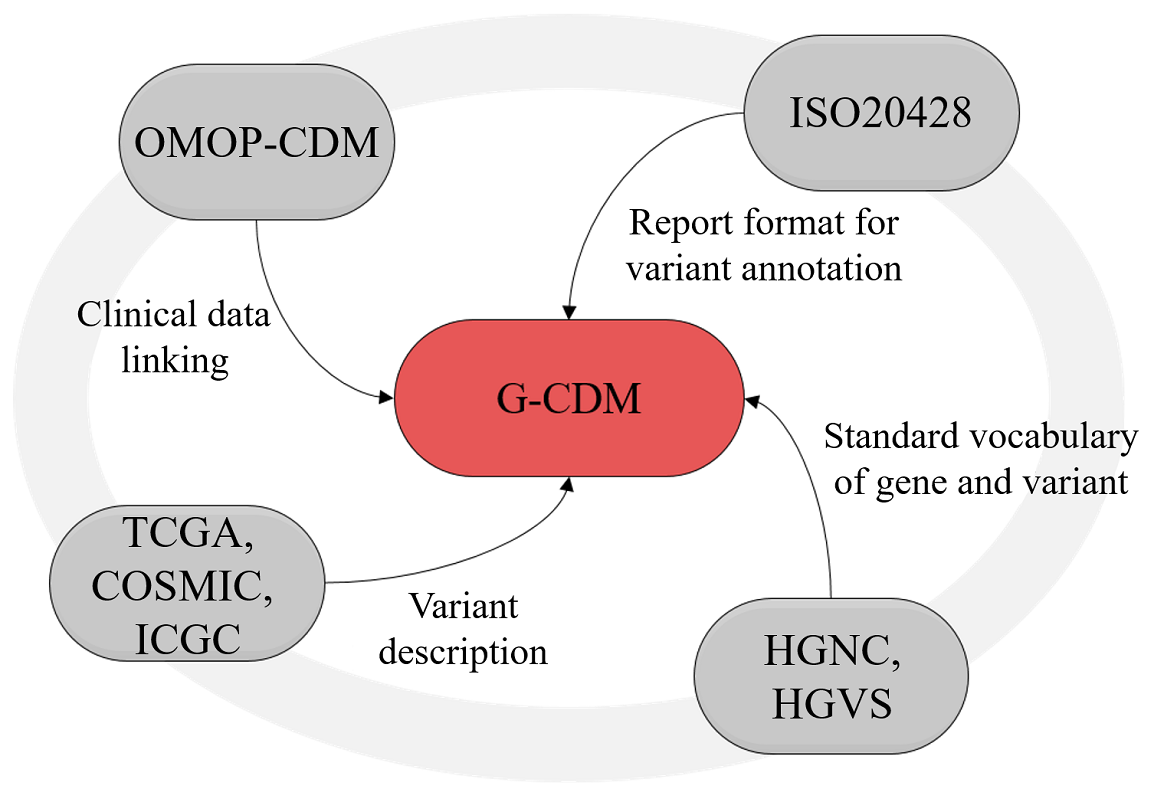

Supplement: Multimedia Appendix 2 [file jmir_v21i3e13249_app2.png]

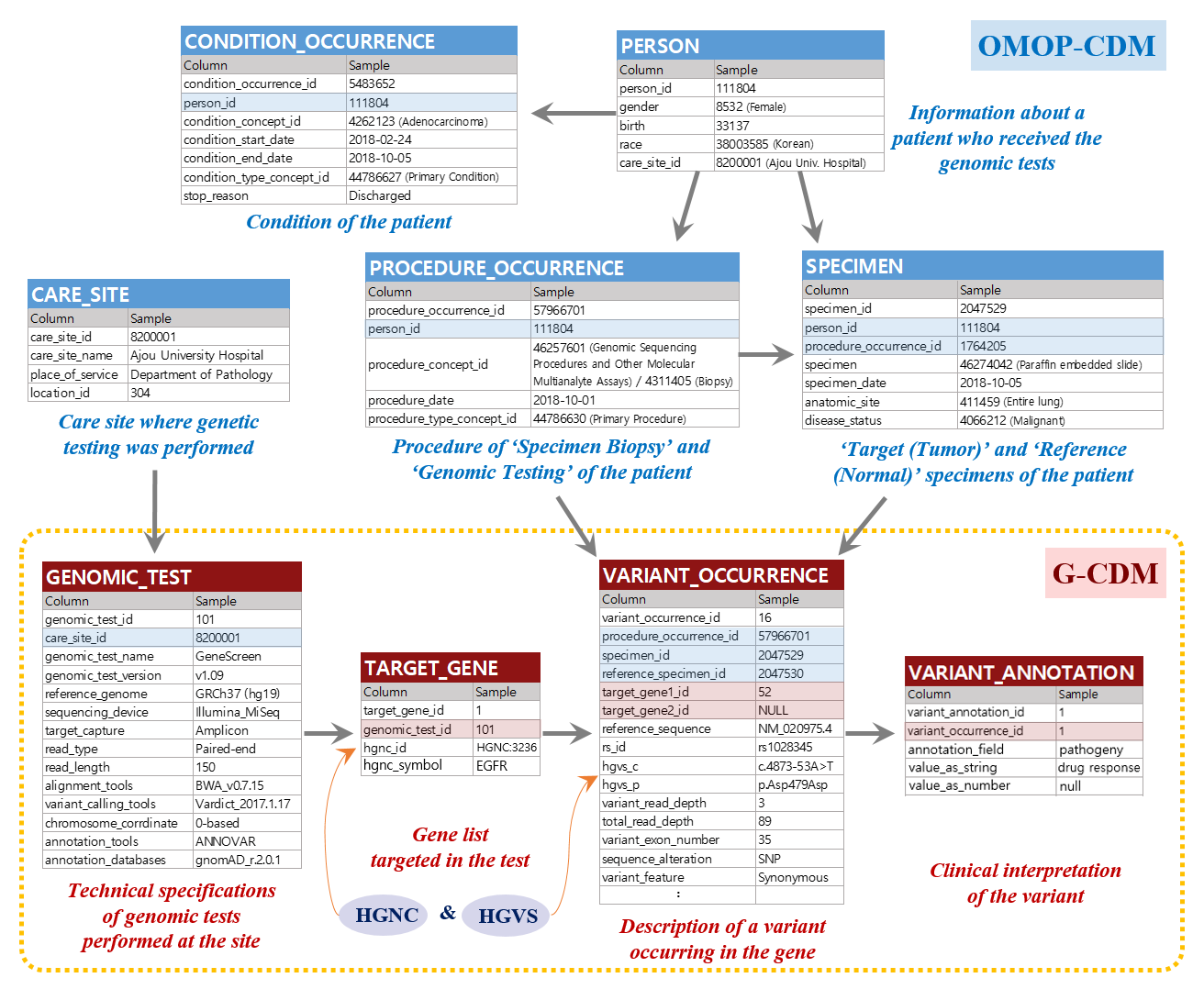

Supplement: Multimedia Appendix 3 [file jmir_v21i3e13249_app3.PNG]

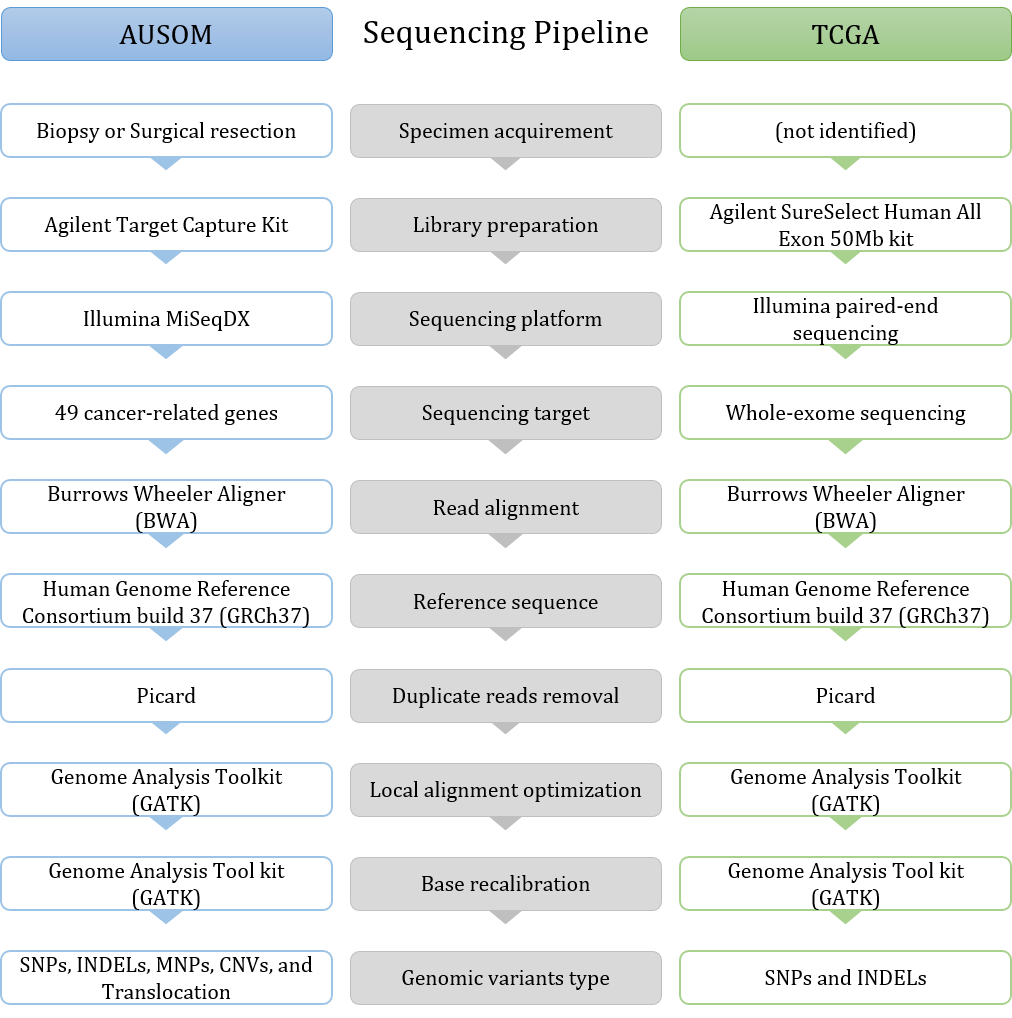

Supplement: Multimedia Appendix 5 [file jmir_v21i3e13249_app5.PNG]

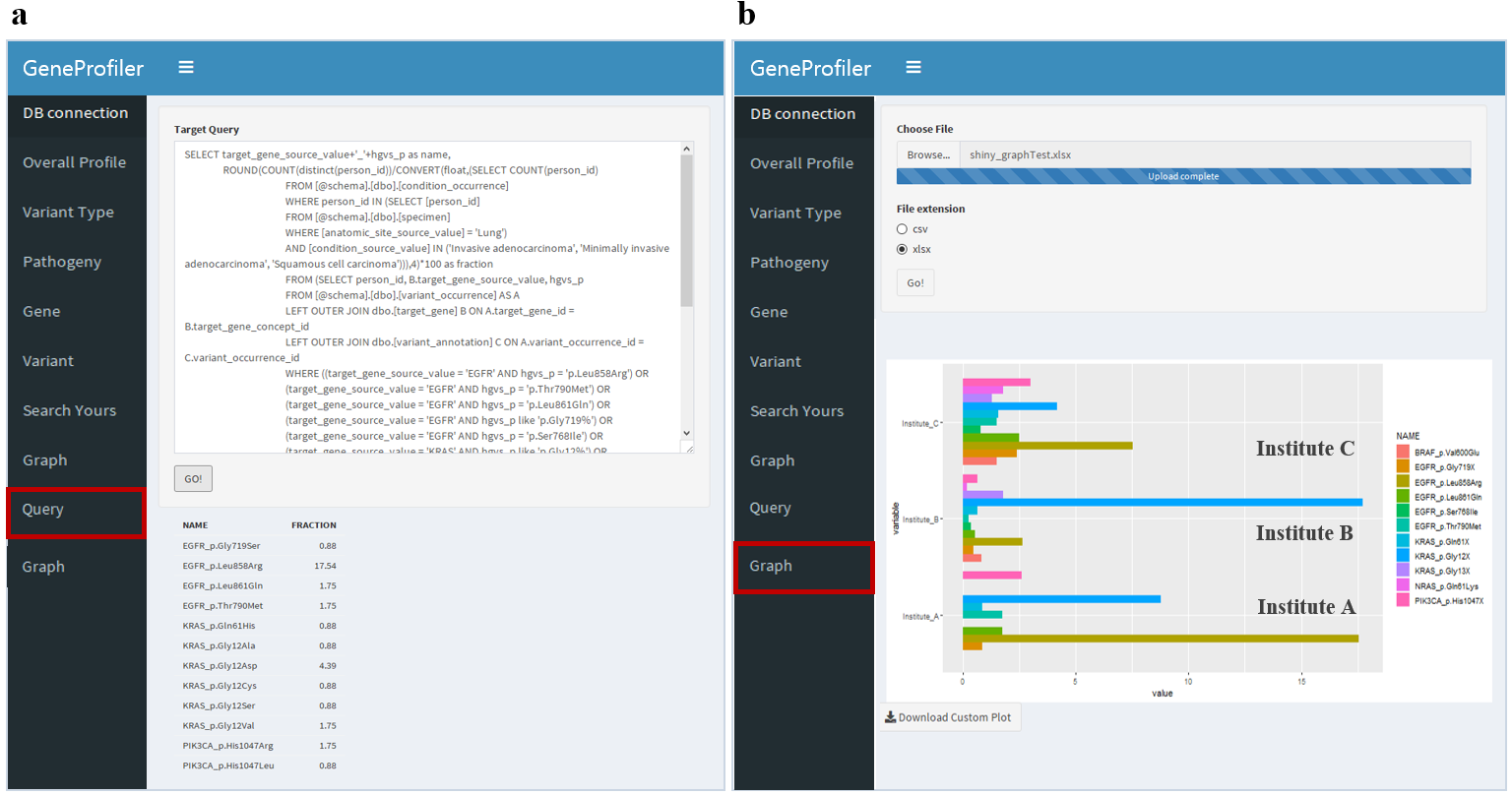

Supplement: Multimedia Appendix 6 [file jmir_v21i3e13249_app6.png]

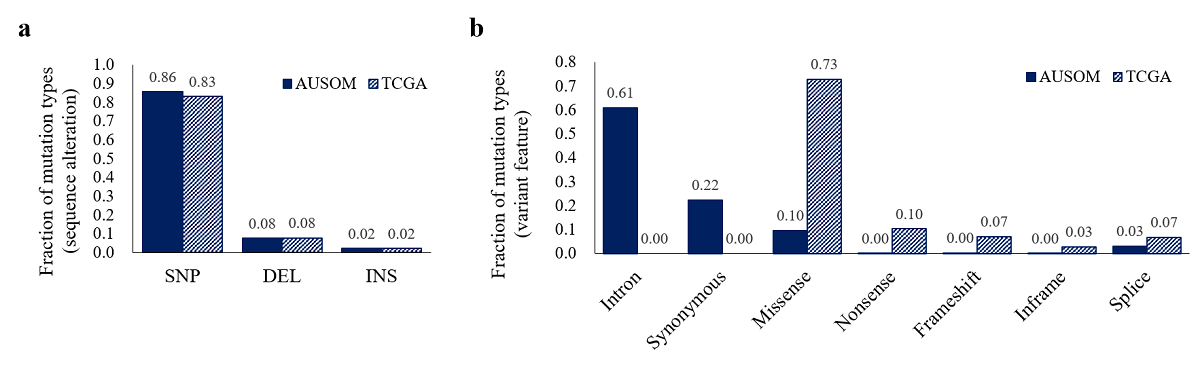

Supplement: Multimedia Appendix 7 [file jmir_v21i3e13249_app7.png]

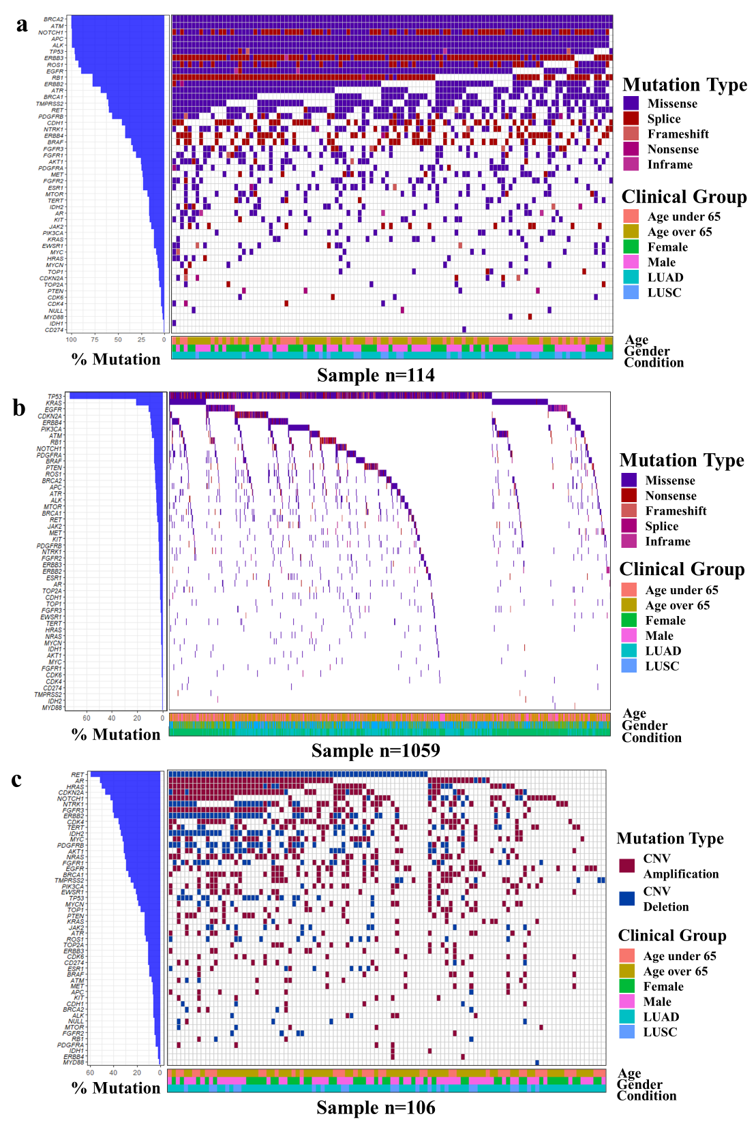

Supplement: Multimedia Appendix 8 [file jmir_v21i3e13249_app8.PNG]
