# Supplementary material for: Genomic Common Data Model for Seamless Interoperation of Biomedical Data in Clinical Practice: Retrospective Study
Source: J Med Internet Res. 2019 Mar 26;21(3):e13249. doi: 10.2196/13249 (PMC6454347; doi:10.2196/13249)
Supplement: Multimedia Appendix 4 [file jmir_v21i3e13249_app4.pdf]

GENOMIC TEST				
#	Field	Required	Type	Description
1	genomic_test_id	Yes	integer	A unique identifier for each platform
2	care_site_id	Yes	integer	A foreign key to the site of primary care in the care_site table, where the details of the care site are stored
3	genomic_test_name	No	varchar(255)	Information about the name of the platform using sequencing assigned by the institution
4	genomic_test_version	No	varchar(50)	Information about the name of the platform using sequencing maintained by the institution
5	reference_genome	No	varchar(50)	Information about the reference genome used for sequencing analysis
6	sequencing_device	No	varchar(50)	Sequencer machine information
7	library_preparation	No	varchar(50)	Information about the preparation method for the sequencing library
8	target_capture	No	varchar(50)	Information about the capture method of the examined and targeted region
9	read_type	No	varchar(50)	Information about the method of sequence reading
10	read_length	No	integer	Information about the length of the read
11	quality_control_tools	No	varchar(255)	Information about the tool used for quality control
12	total_reads	No	integer	Total count of reads involved in the assembly
13	mean_target_coverage	No	float	Mean target coverage
14	per_target_base_cover_100x	No	float	Percentage of selected bases
15	alignment_tools	No	varchar(255)	Information about the name and version of the alignment tool
16	variant_calling_tools	No	varchar(255)	Information about the name and version of the variant calling tool
17	chromosome_corrdinate	No	varchar(255)	Coordinated system for numbering the chromosomes
18	annotation_tools	No	varchar(255)	Information about the tool used for annotation
19	annotation_databases	No	varchar(255)	Information about the database used for annotation

**TARGET GENE**				
#	Field	Required	Type	Description
1	target_gene_id	Yes	integer	A system-generated unique identifier for each target region
2	genomic_test_id	Yes	integer	A foreign key identifier to the platform containing the target region. The details of that platform are stored in the platform_info table.
3	hgnc_id	Yes	varchar(50)	Gene ID based on HGNC nomenclature
4	hgnc_symbol	Yes	varchar(50)	Gene Symbol given by HGNC nomenclauture

VARIANT OCCURENCE				
#	Field	Required	Type	Description
1	variant_occurrence_id	Yes	integer	A unique identifier for each variant occurrence
2	procedure_occurrence_id	Yes	integer	A foreign key identifier to the procedure occurrence table for the procedure used to obtain the specimen
3	specimen_id	Yes	integer	Tumor specimen ID
4	reference_specimen_id	No	integer	ID of normal specimen related to the tumor specimen
5	target_gene1_id	No	varchar(50)	A foreign key identifier to the target_gene table for which the variant information is recorded
6	target_gene2_id	No	varchar(50)	A foreign key identifier to the target_gene table for which the variant information is recorded when a translocation variant occurs
7	reference_sequence	No	varchar(50)	Transcript ID based on a protein-coding RNA (mRNA) made up of the accession number and version number
8	rs_id	No	varchar(50)	dbSNP reference ID maintained by NCBI
9	reference_allele	No	varchar(255)	Reference allele sequence (e.g., A)
10	alternate_allele	No	varchar(255)	Variant allele sequence (e.g., C)
11	hgvs_c	No	varchar(MAX)	Nomenclature for the sequence variant at the DNA level
12	hgvs_p	No	varchar(MAX)	Nomenclature for the sequence variant at the protein level
13	variant_read_depth	No	integer	Variant depth divided by read depth
14	total_read_depth	No	integer	Count of nucleotides involved in the assembly
15	variant_exon_number	No	integer	Exon number in which the variant occurred
16	copy_number	No	float	Copy number value for CNV data
17	cnv_locus	No	varchar(MAX)	Locus information for CNV
18	fusion_breakpoint	No	integer	Sequential position that fusion variant occurred
19	fusion_supporting_reads	No	integer	Supporting read count of the fusion
20	sequence_alteration	No	varchar(MAX)	Structural variant type
21	variant_feature	No	varchar(MAX)	Functional variant type
22	genetic origin	No	varchar(50)	Somatic or germline origin of the variant
23	genotype	No	varchar(50)	Allele state

**VARIANT ANNOTATION**				
#	Field	Required	Type	Description
1	variant_annotation_id	Yes	integer	A unique identifier for each variant annotation event
2	variant_occurrence_id	Yes	integer	A foreign key identifier to the variant_occurrence table for which the variant annotation is recorded
3	annotation_field	Yes	varchar(MAX)	Caterogies or database name of annotation
4	value_as_string	No	varchar(MAX)	Annotation value as a data type of string
5	value_as_number	No	float	Annotation value as a data type of number
